# Head-mounted eye tracker videos and raw data collected during breathing recognition attempts in simulated cardiac arrest

**DOI:** 10.1016/j.dib.2024.110530

**Published:** 2024-05-11

**Authors:** Marco Pedrotti, Marc Stanek, Louis Gelin, Philippe Terrier

**Affiliations:** Haute École Arc Santé, HES-SO University of Applied Sciences and Arts Western Switzerland, Espace de l'Europe 11, 2000 Neuchâtel, Switzerland

**Keywords:** Eye tracking, Eye movements, Gaze point, Pupil diameter, Head-mounted eye tracker, Video, Cardiac arrest, Cardiopulmonary resuscitation

## Abstract

This paper presents data collected by Pedrotti et al. (2022, 2024) [1,2], which includes videos captured using a Dikablis head-mounted eye tracker (Ergoneers GmbH, Germany), along with the corresponding raw data. The data collection aimed to assess participants' ability to recognize breathing in a simulated cardiac arrest scenario. Equipped with the eye tracker, participants entered a room where a manikin was positioned on the floor. Their task was to determine if the manikin was breathing and respond accordingly, such as initiating cardiopulmonary resuscitation if the victim was not breathing. Our analysis focused on examining looking time on the manikin's thorax by inspecting the videos. Potential applications of the dataset [3] include identifying fixation and saccades using custom algorithms, analyzing pupil diameter data, and conducting secondary analyses involving participant characteristics like age and gender as independent variables.

Specifications Table

**Table:** 

Subject	Emergency Medicine
Specific subject area	First Aid Education
Type of data	- Videos (.mp4) in 1st person view, filmed with the head-mounted eye tracker scene camera - Binocular eye tracking raw data (.txt) sampled at 120Hz: gaze point and pupil diameter - Table (.xlsx) containing participant demographics, experimental conditions, and the thorax observation time that we computed by reviewing the videos
Data collection	Participants wearing a head-mounted eye tracker entered a room where a manikin, randomly assigned as either breathing or not, was positioned on the floor. Their task was to assess whether CPR was necessary (for an unresponsive and non-breathing victim) or if the recovery position was appropriate (for an unresponsive and breathing victim), and to determine the appropriate course of action. Following the initial simulation (pre-allocation), participants were randomly assigned to either an experimental group, receiving video debriefing with their recorded gaze overlay, or a control group without this overlay. After debriefing, the simulation was repeated (post-allocation).
Data source location	· Institution: Haute École Arc, Campus Arc 2 · City/Town/Region: Neuchâtel · Country: Switzerland · Latitude and longitude for collected samples/data: Latitude: 46.9970672; Longitude: 6.9395648
Data accessibility	Repository name: Zenodo Data identification number: 10,952,880 Direct URL to data: https://doi.org/10.5281/zenodo.10952880
Related research article	M. Pedrotti, M. Stanek, L. Gelin, P. Terrier, Replication Study of “Visual Fixation on the Thorax Predicts Bystander Breathing Detection in Simulated Out-of-Hospital Cardiac Arrest”, Int. J. First Aid Education, 7 (2024). https://doi.org/10.25894/ijfae.2307

## 1 Value of the Data

• The videos are valuable resources for researchers focusing on first aid, Basic Life Support (BLS), and cardiopulmonary resuscitation (CPR), as well as for trainers conducting first aid, BLS and CPR courses. Additionally, the raw data can be utilized for the development and evaluation of fixation/saccade detection algorithms.
• Potential applications of the data include (but are not restricted to) analyzing pupil diameter data, employing custom algorithms to identify fixations and saccades, and conducting secondary analyses (e.g., using participant demographics as independent variables).
• Making raw data publicly available facilitates independent replication and validation of our results, thereby increasing the overall robustness and confidence in research findings.

## 2 Background

Out-of-Hospital Cardiac Arrest (OHCA) remains a significant global cause of mortality, emphasizing the critical need for prompt intervention [4]. Resuscitation guidelines stress the importance of initiating cardiopulmonary resuscitation (CPR) upon encountering an unresponsive individual with absent or irregular breathing [5,6], as it is strongly associated with improved patient outcomes [7]. Recognizing OHCA, characterized by unresponsiveness and lack of breathing in the victim, is crucial for initiating CPR without delay. In our studies, we conducted experiments to explore the relationship between the duration of looking at the thorax and the ability to recognize OHCA. Study 1 [1] represents the initial investigation, while Study 2 [2] serves as a replication of Study 1, employing the same methods.

This data article supplements the published research by providing a comprehensive overview of the raw data, which is publicly accessible in a data repository. Additionally, it offers insights into potential applications and reuse of this data, further contributing to our understanding of OHCA recognition and response.

## 3 Data Description

The Excel file ``*data summary.xlsx*'' comprises participant demographics, details on experimental conditions, and the thorax observation duration derived from video analysis (analyzed data). Table 1 provides a list of variable names along with corresponding explanations of their significance.

**Table 1:** Variable names and significance of “data summary.xlsx”.

Study	The data were collected in 2 studies using the same methods. Study 1 is the original study (https://doi.org/10.1097/SIH.0000000000000617), Study 2 is the replication study (https://doi.org/10.25894/ijfae.2307)
ID	Participant identification number
Gender Female=1	Participant gender. ``1” stands for ``female''. ``0” stands for "male"
Date	Date (dd.mm.yyyy) at which the simulation occurred
Age	Age (years)
Height (m)	Height (meters)
Mass (kg)	Body weight (kilograms)
BMI	Body Mass Index
Glasses yes=1	Whether the participant usually wears glasses. ``1” stands for ``yes''. ``0” stands for ``no''
Lenses yes=1	Whether the participant usually wears contact lenses. ``1” stands for ``yes''. ``0” stands for ``no''
First aid course driver's license yes =1	Whether the participant had taken a first aid course as part of his/her driving licence procedure. ``1” stands for ``yes''. ``0” stands for ``no''
Group experimental=1	Experimental group to which the participant belongs. ``1” stands for ``experimental group''. ``2” stands for ``control group''
Condition breathing=1	Whether the manikin had been set to ``breathing'' or ``no breathing''. ``1” stands for ``breathing''. ``0” stands for ``no breathing''
Result success=1	Whether the participant made the right action, i.e. placed a breathing manikin in the recovery position or started CPR on a non-breathing manikin. ``1” stands for ``success''. ``0” stands for ``failure''
Time first visual contact	The time (in seconds) at which the participant first looked at the manikin
Time rescue action	The time (in seconds) at which the participant started the recovery action (recovery position or CPR) of his/her choice
Time to acting	Difference between ``Time rescue action'' and ``Time first visual contact''
Thorax observation start	The time (in seconds) at which the participant first looked at the manikin's thorax
Thorax observation end (decision)	The time (in seconds) at which the participant stopped looking at the manikin's thorax
Thorax observation time	Difference between ``Thorax observation end (decision)'' and ``Thorax observation start''

The two .zip files “Study 1.zip” and “Study 2.zip” contain videos (.mp4) and raw data (.txt) for Study 1 and Study 2 respectively. Each zip file contains two folders named “eye tracking video” and “eye tracking data”. File names start with participant ID number followed by the experimental condition (e.g. “6 preallocation.txt”, “47 postallocation.mp4”; see “Data collection” above).

The file “readme.txt” contains basic information required by Zenodo and is self-explanatory.

## 4 Experimental Design, Materials and Methods

### 4.1 Study design

A prospective, blinded, single-center, 2-arm parallel randomized controlled trial was conducted with balanced randomization (1:1). The trial aimed to assess the superiority of a novel intervention, specifically video debriefing with or without eye-tracking gaze overlay. The trial protocol consisted of three main stages: pre-allocation simulation, intervention (video debriefing with or without gaze overlay), and post-allocation simulation.

The initial trial (Study 1) was followed by a subsequent trial (Study 2) with the objective of replicating the findings.

### 4.2 Participants

Participants were eligible for inclusion if they met the following criteria:

1. Enrollment in the Healthcare Propaedeutic Year (HPY), which encompasses healthcare theory, hands-on classes, and internships in healthcare institutions. Completion of the HPY is a prerequisite for enrollment in the Bachelor of Nursing program.

2. Attainment of the "Basic Life Support–Automated External Defibrillation–Swiss Resuscitation Council (BLS-AED-SRC)" certification during the HPY.

The mean age of participants was 21 years (SD = 4) for Study 1 and 21 years (SD = 3) for Study 2. In both studies, 77 % of participants were female.

### 4.3 Procedure

Wearing an eye tracker, participants entered a room where a manikin was placed on the floor, randomly set as either breathing or unbreathing. They were assigned the responsibility of deciding whether CPR was necessary for the victim in instances of unresponsiveness and absence of breathing, or if placing them in a recovery position was warranted if they were unresponsive yet still breathing. Following the initial simulation (pre-allocation), participants were randomly assigned to either an experimental group, receiving video debriefing with their recorded gaze overlay, or a control group without the gaze overlay. During the debriefing session, a certified trainer focused on the thorax examination behavior captured during the pre-allocation phase. After debriefing, participants repeated the simulation (post-allocation) [1][2]. The primary outcome measured success in identifying breathing, indicated by initiating CPR for an unbreathing manikin or placing a breathing manikin in a recovery position. Secondary outcome measures included thorax examination time, reflecting the cumulative duration spent observing the manikin's chest.

## Limitations

In Study 1, out of the initial 125 participants, 28 were unable to complete or even start the study due to technical issues with eye tracking, such as calibration difficulties or pupil detection failure. This resulted in a data loss of 22.4 %.

In Study 2, among the initial 84 participants, 11 were unable to complete or start the study due to technical eye tracking problems, accounting for a data loss of 13.1 %. Additionally, valid data for the secondary outcome (thorax examination time) were obtained only from 52 participants due to poor recording quality of eye tracking.

## Ethics Statement

The research adhered to the principles outlined in the Declaration of Helsinki. The study protocol underwent review by Swissethics, who determined that it did not fall within the scope of the Federal Act on Research involving Human Beings [8]. The study was conducted in compliance with the Federal Act on Data Protection [9], and all participants provided signed informed consent forms.

### Declaration of generative AI and AI-assisted technologies in the writing process

During the preparation of this work the authors used Deepl and ChatGPT 3.5 to translate from French to English. After using these tools, the authors reviewed and edited the content as needed and take full responsibility for the content of the publication.

## CRediT authorship contribution statement

**Marco Pedrotti:** Conceptualization, Methodology, Validation, Formal analysis, Investigation, Data curation, Writing – original draft, Writing – review & editing, Visualization, Supervision, Project administration, Funding acquisition. **Marc Stanek:** Conceptualization, Investigation, Writing – review & editing. **Louis Gelin:** Conceptualization, Investigation, Writing – review & editing. **Philippe Terrier:** Conceptualization, Methodology, Software, Validation, Formal analysis, Investigation, Data curation, Writing – review & editing, Visualization.

## Data Availability

Eye tracking videos and raw data of breathing recognition attempts in simulated out-of-hospital cardiac arrest (Original data) (Zenodo)
